# Novel *RPL13* Variants and Variable Clinical Expressivity in a Human Ribosomopathy With Spondyloepimetaphyseal Dysplasia

**DOI:** 10.1002/jbmr.4177

**Published:** 2020-10-13

**Authors:** Alice Costantini, Jessica J Alm, Francesca Tonelli, Helena Valta, Céline Huber, Anh N Tran, Valentina Daponte, Nadi Kirova, Yong‐Uk Kwon, Jung Yun Bae, Woo Yeong Chung, Shengjiang Tan, Yves Sznajer, Gen Nishimura, Tuomas Näreoja, Alan J Warren, Valérie Cormier‐Daire, Ok‐Hwa Kim, Antonella Forlino, Tae‐Joon Cho, Outi Mäkitie

**Affiliations:** ^1^ Department of Molecular Medicine and Surgery and Center for Molecular Medicine Karolinska Institutet Stockholm Sweden; ^2^ Department of Molecular Medicine, Biochemistry Unit University of Pavia Pavia Italy; ^3^ Children's Hospital, Pediatric Research Center University of Helsinki and Helsinki University Hospital Helsinki Finland; ^4^ Department of Clinical Genetics INSERM UMR 1163, Paris Descartes‐Sorbonne Paris Cité University, Imagine Institute, Necker Enfans Malades Hospital (AP‐HP) Paris France; ^5^ Division of Pathology, Department of Laboratory Medicine Karolinska Institutet Huddinge Sweden; ^6^ Department of Orthopaedic Surgery Busan Paik Hospital, Inje University College of Medicine Busan South Korea; ^7^ Department of Orthopaedic Surgery Pusan National University Yangsan Hospital, Pusan National University School of Medicine Yangsan Republic of Korea; ^8^ Department of Pediatrics Busan Paik Hospital, College of Medicine, Inje University Busan Republic of Korea; ^9^ Cambridge Institute for Medical Research, Keith Peters Building Cambridge UK; ^10^ Department of Haematology University of Cambridge Cambridge UK; ^11^ Wellcome Trust–Medical Research Council Stem Cell Institute, University of Cambridge Cambridge UK; ^12^ Centre de Génétique Humaine – CGH Cliniques Universitaires St. Luc, UCL Bruxelles Belgium; ^13^ Center for Intractable Diseases Saitama Medical University Hospital Saitama Japan; ^14^ Department of Radiology, I‐Bone Hospital Cheonan Republic of Korea; ^15^ Division of Pediatric Orthopaedics, Seoul National University Children's Hospital Seoul Republic of Korea; ^16^ Department of Clinical Genetics Karolinska University Hospital Stockholm Sweden; ^17^ Folkhälsan Institute of Genetics, and Research Program for Clinical and Molecular Metabolism, University of Helsinki Helsinki Finland

**Keywords:** CRISPR‐CAS9, INCOMPLETE PENETRANCE, RIBOSOMOPATHY, RPL13, SPONDYLOEPIMETAPHYSEAL DYSPLASIA, VARIABLE EXPRESSIVITY, ZEBRAFISH

## Abstract

Spondyloepimetaphyseal dysplasias (SEMDs) are a heterogeneous group of disorders with variable growth failure and skeletal impairments affecting the spine and long bone epiphyses and metaphyses. Here we report on four unrelated families with SEMD in which we identified two monoallelic missense variants and one monoallelic splice site variant in *RPL13*, encoding the ribosomal protein eL13. In two out of four families, we observed autosomal dominant inheritance with incomplete penetrance and variable clinical expressivity; the phenotypes of the mutation‐positive subjects ranged from normal height with or without hip dysplasia to severe SEMD with severe short stature and marked skeletal dysplasia. *In vitro* studies on patient‐derived dermal fibroblasts harboring *RPL13* missense mutations demonstrated normal eL13 expression, with proper subcellular localization but reduced colocalization with eL28 (*p* < 0.001). Cellular functional defects in fibroblasts from mutation‐positive subjects indicated a significant increase in the ratio of 60S subunits to 80S ribosomes (*p* = 0.007) and attenuated global translation (*p* = 0.017). In line with the human phenotype, our *rpl13* mutant zebrafish model, generated by CRISPR‐Cas9 editing, showed cartilage deformities at embryonic and juvenile stages. These findings extend the genetic spectrum of *RPL13* mutations causing this novel human ribosomopathy with variable skeletal features. Our study underscores for the first time incomplete penetrance and broad phenotypic variability in SEMD‐RPL13 type and confirms impaired ribosomal function. Furthermore, the newly generated *rpl13* mutant zebrafish model corroborates the role of eL13 in skeletogenesis. © 2020 The Authors. *Journal of Bone and Mineral Research* published by Wiley Periodicals LLC on behalf of American Society for Bone and Mineral Research (ASBMR)..

## Introduction

Osteochondrodysplasias, also known as skeletal dysplasias, are a heterogeneous group of genetic diseases affecting development of bone and cartilage. More than 450 different forms have so far been recognized and classified based on radiological or molecular features.^(^ ^1^ ^)^ Spondyloepimetaphyseal dysplasias (SEMDs) are characterized by severe short stature and major skeletal abnormalities in the spine, epiphyses, and metaphyses. Nowadays SEMDs are classified in 19 different subtypes.^(^ ^1^ ^)^ Despite phenotypic similarities, the underlying molecular defects cover diverse biological processes ranging from extracellular matrix composition^(^ ^2^, ^3^, ^4^ ^)^ to cell signaling.^(^ ^5^, ^6^ ^)^

Recently, Le Caignec and colleagues reported monoallelic *de novo* mutations in the gene encoding the ribosomal protein eL13 (*RPL13*) in four patients with SEMD.^(^ ^7^ ^)^ This finding expands the number of ribosomopathies, a group of congenital diseases defined by mutations in genes encoding ribosomal proteins (RPs), transcribing ribosomal RNAs (rRNAs), or factors involved in ribosome biogenesis.^(^ ^8^, ^9^ ^)^ Ribosomopathies, which often feature a broad phenotypic variability, include for instance Diamond‐Blackfan anemia (MIM #105650), Shwachman‐Diamond syndrome (MIM #260400), Treacher Collins syndrome (MIM #154500), and cartilage‐hair hypoplasia (MIM #250250).^(^ ^10^, ^11^, ^12^, ^13^, ^14^, ^15^ ^)^ These diseases are most commonly characterized by defects in the hematopoietic system and the skeleton, but the phenotypic presentation varies between disorders, among individuals with the same disorder and even among family members sharing identical gene defects.^(^ ^8^, ^9^, ^16^ ^)^

eL13 is an integral component of the large (60S) ribosomal subunit that is required for pre‐ribosomal RNA processing.^(^ ^7^ ^)^ Despite the fact that eukaryotic ribosomes (80S) are essential for cell growth and proliferation, it is still largely unclear why mutations in *RPL13* and other genes partaking in ribosome biogenesis lead to tissue‐specific consequences. Furthermore, molecular mechanisms underlying ribosome dysfunction remain inadequately explored.

As part of our research program on genetic causes of osteochondrodysplasia, we recruited four unrelated families in which the index patients featured SEMD with a severe growth disorder and uniform radiological features. In each of these families, we identified an *RPL13* mutation and studied the disease pathogenesis by performing both *in vitro* and *in vivo* studies. Zebrafish was chosen as our disease model not only because 71% of the genome is conserved between humans and this teleost fish, but also because zebrafish is a proven model for investigating skeletal diseases during development,^(^ ^17^, ^18^, ^19^ ^)^ as bone development and some skeletal components are highly conserved between these two species.^(^ ^20^ ^)^

## Materials and Methods

### Subjects

Our study included one Finnish index patient (patient 1), two Korean index patients (patients 2 and 3), and one French/Congolese patient (patient 4), all affected by an unusual form of SEMD. Their parents and other family members, some of whom were possibly affected, were also enrolled in the study. Clinical data and radiographs were collected from medical records and reviewed for disease features and skeletal characteristics. All subjects were also clinically examined as part of this research. Unrelated healthy controls were included for functional validations on dermal fibroblasts.

### Whole‐genome and exome sequencing

To identify the genetic defect underlying severe skeletal dysplasia in families 1 to 4, we adopted different strategies. The genetic cause of disease was first characterized in families 1 and 4 by performing massively parallel sequencing (MPS).

In family 1, we performed trio whole‐genome sequencing (WGS) as previously described.^(^ ^21^, ^22^ ^)^ For data analysis, we applied the following filtering criteria: (i) homozygous/compound heterozygous variant or *de novo* variant; (ii) MAF <0.001 the gnomAD^(^ ^23^ ^)^ and SweGen^(^ ^24^ ^)^ databases; and (iii) impact severity other than LOW in GEMINI.^(^ ^25^ ^)^

In family 4, the index patient underwent whole‐exome sequencing analysis (WES), which was carried out using our previously described in‐house pipeline.^(^ ^26^ ^)^

Further information about these two methods are included in Supplemental Materials and Methods.

### Sanger sequencing

To identify the genetic cause of the skeletal disease in families 2 and 3, we screened for mutations the candidate gene identified in families 1 and 4 using Sanger sequencing, as previously described.^(^ ^21^ ^)^ This method was also chosen to validate the MPS findings in families 1 and 4. Primer sequences are available from the authors upon request.

### Molecular modeling

The PyMOL Molecular Graphics System, Version 2.0.7 (Schrödinger, LLC, New York, NY, USA) was used to generate atomic models based on human ribosome structures derived by cryo‐electron microscopy (pdb codes: 6EK0 and 4V6X)^(^ ^27^, ^28^ ^)^ and to simulate *in silico* the effects of the identified mutations on protein folding.

### Dermal fibroblast cultures

Skin biopsies were harvested from four mutation‐positive subjects (patients 1, 2, and 3 and the affected mother of patient 2) and five controls (both parents of patient 1 and three unrelated controls). Fibroblasts were isolated as previously described,^(^ ^29^ ^)^ and cultures expanded in high‐glucose DMEM (Thermo Fisher Scientific, Waltham, MA, USA; cat. #11960044) supplemented with 1% GlutaMAX (Thermo Fisher Scientific; cat. #35050038), antibiotics (penicillin and streptomycin at a final concentration of 100 U/mL), and 15% fetal bovine serum. Media were changed every 3 to 4 days, and cells were split upon 80% confluence. Cells of passage 3 to 6 were used for characterization assays. See Supplemental Materials and Methods for details.

### Total protein extraction and Western immunoblotting

For characterization of the fibroblasts at the protein level, cells were grown to 90% confluence in 100 mm dishes and lysed in ice‐cold RIPA‐buffer containing protease inhibitors. Cell lysate was collected by centrifugation followed by total protein quantification (BCA kit according to manufacturer's protocol). Expression of eL13 was evaluated in 10 μg of denatured protein samples by Western immunoblotting (WB) using a primary antibody against RPL13 (mouse monoclonal, Santa Cruz Biotechnology, Dallas, TX, USA; #sc‐100829; 1:1,000 dilution) and a HRP‐conjugated anti‐mouse secondary antibody (Thermo Fisher Scientific; #31430, 1:20,000 dilution) according to standard procedures. Acetylated‐tubulin (Sigma‐Aldrich, St. Louis, MO, USA; #T7451, 1:10,000 dilution) was used as a loading control. Further information is available in the Supplemental Materials and Methods.

### Immunocytochemistry

To investigate possible effects of the mutations on the colocalization of eL13 with other RPs, fibroblasts were cultured on glass cover slips supplemented with 100 μM ascorbic acid 2‐phosphate to stimulate extracellular matrix (ECM) production. This culture condition was chosen to put cells under higher protein synthesis demands, which could emphasize possible alterations in patient‐derived cells. Immunocytochemistry was performed using standard procedures (detailed information in Supplemental Materials and Methods and in Supplemental Table S1). For colocalization analysis, eL13 was co‐stained with eL7, eL28, eS19, and calnexin, respectively. Cover slips (3/donor/staining) were imaged with a NikonA1+ confocal laser microscope system. Z‐stacks were captured using 60× objective fulfilling Nyquist sampling theorem. Laser power and detector gain were adjusted to cover the widest possible range of intensity values for colocalization analysis. From the entire Z‐stacks, 3D colocalization was measured per cell as Pearson's correlation coefficient using the Colocalization Test plugin in ImageJ Fiji.^(^ ^30^ ^)^ Pictures shown are z‐stacks processed to maximum intensity projections. See Supplemental Materials and Methods for further details.

### Sucrose density gradients

Sucrose density gradient fractionation of cell extracts was performed, with minor modifications, as previously described.^(^ ^31^, ^32^, ^33^ ^)^ Refer to the Supplemental Materials and Methods for a detailed description of the method.

### Measurement of protein synthesis

Protein synthesis was measured as described by Tan and colleagues^(^ ^33^ ^)^ and in the Supplemental Materials and Methods.

### Zebrafish husbandry

Wild‐type (AB) zebrafish (*Danio rerio*), obtained from the European Zebrafish Resource Center (EZRC; Eggenstein‐Leopoldshafen, Germany), were housed in the animal facility of the University of Pavia in a ZebTEC semi‐closed recirculation housing system (Tecniplast, Buguggiate, Italy) at 28°C, pH 7.5, and conductivity 500 μS on a 14/10 light/dark cycle. Fish were fed three times a day alternating dry food and brine shrimps (Zebrafish Management Ltd., Twyford, UK). Embryos were kept in petri dishes in fish water (NaHCO_3_ 1.2 mM, instant ocean 0.1 g/L, CaSO_4_ 1.4 mM, methylene blue 0.00002% w/v) at 28°C. For experiments, embryos and juveniles were anesthetized using 0.016% w/v tricaine (3‐amino benzoic acidethylester, Sigma‐Aldrich, Darmstadt, Germany) in zebrafish water and euthanized by tricaine overdose (0.03% w/v).

### The mutant *rpl13* CRISPR‐Cas9 zebrafish line

To investigate the role of eL13 in skeletal development *in vivo*, a zebrafish model harboring a homozygous mutation in *rpl13*, hereafter called “*rpl13* mutant”, was generated. *rpl13* (transcript ID: ENSDART000001168460.2), the ortholog of the human *RPL13*, was targeted by CRISPR‐Cas9 gene editing. The guide RNA (gRNA) was designed using the free online software CHOPCHOP (https://chopchop.rc.fas.harvard.edu)^(^ ^34^, ^35^ ^)^ to target exon 6 (5′‐GGCACGGATGCCGAAAAGACGGG‐3′). Details on cloning, transcription of gRNA and Cas9, embryo‐injection, and mutation screening are described in Supplemental Materials and Methods.

### Transcript and protein analysis in the *rpl13* mutant zebrafish

To evaluate the expression of *rpl13* at the RNA and protein levels in zebrafish, quantitative PCR (qPCR) and WB were performed. Detailed information about these methods can be found in Supplemental Materials and Methods.

### Morphometric analysis of the *rpl13* mutant zebrafish

For studying fish morphology, zebrafish embryos at 5 days post fertilization (dpf) (wild‐type, WT *n* = 23, *rpl13* mutant *n* = 10), at 7 dpf (WT *n* = 34, *rpl13* mutant *n* = 47), and juvenile fish at 1 month post fertilization (mpf) (WT *n* = 37, *rpl13* mutant *n* = 22) were anesthetized with tricaine and images were acquired with a Leica (Buffalo Grove, IL, USA) M165 FC stereomicroscope connected to a Leica DFC425 C digital camera. The Leica LAS v4.5 software was used to evaluate the standard length (SL), the height at the anterior margin of the anal fin (HAA), the snout‐operculum length (SOL), and the height at eye (HE) as previously described.^(^ ^36^, ^37^ ^)^ In addition, the level of inflation of the swim bladder was evaluated by counting the numbers of no (0), partially (0.5), or fully (1 or 2) inflated lobes on lateral transmission images at 5 (WT *n* = 23, *rpl13* mutant *n* = 10), 7 dpf (WT *n* = 34, *rpl13* mutant *n* = 47), and 1 mpf (WT *n* = 38, *rpl13* mutant *n* = 22).

### Cartilage staining of the *rpl13* mutant zebrafish

To investigate cartilage development, after euthanasia, 5 dpf (WT *n* = 21, *rpl13* mutant *n* = 9), 7 dpf (WT *n* = 32, *rpl13* mutant *n* = 33), and 1 mpf (WT *n* = 34, *rpl13* mutant *n* = 20) zebrafish were fixed overnight in 4% (w/v) paraformaldehyde (PFA, Merck KGaA, Darmstadt, Germany) at 4°C and stained in 0.02% (w/v) Alcian blue (Sigma‐Aldrich) as previously described.^(^ ^17^ ^)^ Images were acquired on ventral orientation using M165 FC stereomicroscope (Leica) connected to DFC425C digital camera (Leica). The presence of cartilage deformities was investigated by measuring the following parameters: the angle of the ceratohyal (CH) cartilage, the width of Meckel's cartilage (MK), which is the distance between the two opposite sites of MK, and the distance between the tip of CH and the tip of MK.^(^ ^38^ ^)^ To investigate body disproportions in the head and body of the fish at 1 mpf, the SL/HAA and SOL/HE ratios were analyzed. Measurements were performed using the LAS v4.5 (Leica) software.

### Statistics

Data analyses were performed using two‐tailed statistical tests. Results from qPCR and densitometries of WBs are presented as mean ± SD and analyzed using Student's *t* test. Colocalization analyses in fibroblasts were quantified as Pearson's correlation coefficient from z‐stacks of three samples/donor/staining and analyzed using non‐parametric Mann–Whitney *U* test in SPSS (IBM Inc., Armonk, NY, USA) because the data did not fulfill criteria of normal distribution or equal variances. Mann–Whitney *U* test was also used to analyze the data from the protein synthesis assay because the data were not normally distributed. Because of the limited sample size of the control group (*n* = 2) from sucrose profiling, data from mutation‐positive cells were compared against the median (0.5205) of the control group using one‐sample *t* test. Zebrafish data are presented as following. SL, distance between the tip of CH and the tip of MK, width of MK and CH angle in the fish larvae at 5 and 7 dpf were analyzed by Student's *t* test. Statistics for swim bladder inflation was performed as frequency analysis of number of inflated lobes (0, 0.5, 1, or 2 lobes) using crosstabs with chi‐square test. Data concerning SL, distance between the tip of CH and the tip of MK, and width of MK in fish at 1 mpf were divided in categories based on global quartiles and analyzed using chi‐square test. The same strategy was applied for analyzing SL/HAA and SOL/HE ratios, where frequencies were analyzed using chi‐square test after categorizing based on global quartiles. All data are presented as means/medians with interquartile ranges. A *p* value <0.05 was considered significant.

### Study approval

All studies were conducted in accordance with the Declaration of Helsinki.^(^ ^39^ ^)^ Research protocols were approved by the Institutional Ethics Committees of the Helsinki University Hospital, the Necker Enfants Malades Hospital in Paris, and the Seoul National University Children's Hospital. A written informed consent was signed by all participants and/or their caregivers before inclusion and sample collection. The zebrafish experiments were performed in agreement with EU Directive 2010/63/EU for animals, and the animal protocol was approved by the Italian Ministry of Health (approval no. 1191‐2016‐PR).

## Results

### Similar skeletal features but variable severity in the four index patients

**Fig 1:**
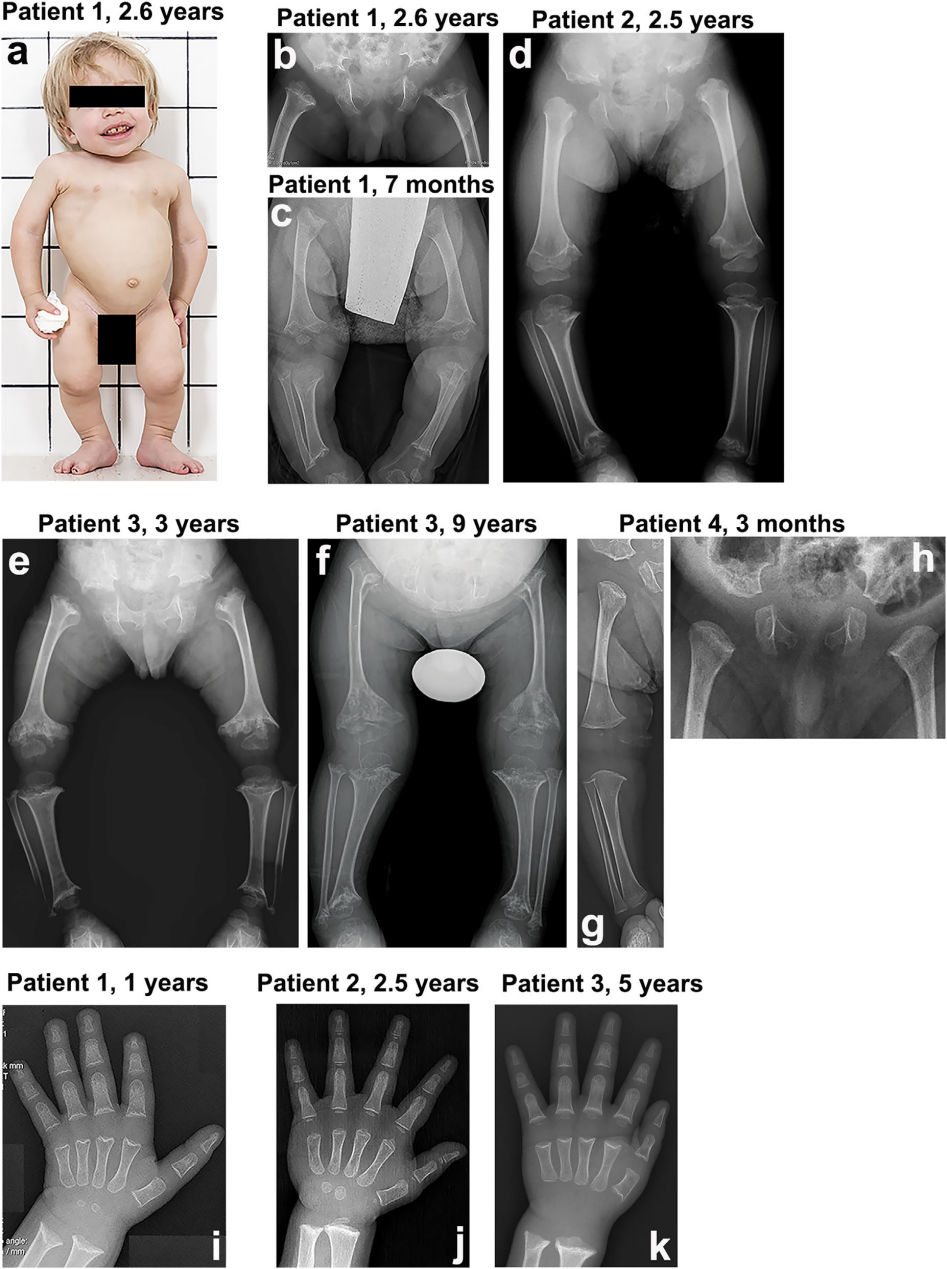
Clinical and radiological features of the four index patients with spondyloepimetaphyseal dysplasia. (*A*) Patient 1 at 2.6 years. The clinical features include mild coarseness of facial features with short nose, depressed nasal bridge and hypomineralization of the teeth, severe short stature with short neck, trunk, and limbs, narrow thorax, protruding abdomen, and flexion contractures of the hip and knee joints. Pelvis and lower limbs in patient 1 (*B*, *C*), patient 2 (*D*), patient 3 (*E*, *F*), and patient 4 (*G*, *H*). The iliac bones are short and broad with serrated iliac crests. The acetabulae are flat and wide with spur‐like projections at the medial and lateral margins. The ischia and pubes are thick with serration at the lateral margin of the ischia. The femora are mildly bowed. The metaphyses of the long bones are wide, cupped, and severely irregular (arrows). The capital femoral epiphyses display significantly delayed ossification, while the other epiphyses of the long bones show normal maturation. The epimetaphyseal changes in patient 3 progressively worsened with age (*E*, *F*). Megaepiphyses are found at the knee and ankle. Wrist and hand in patient 1 (*I*), patient 2 (*J*), and patient 3 (*K*). Metaphyseal cupping and irregularity of the radius and ulna are apparent in patients 1 and 3, while those are mild in patient 2. Metaphyseal cupping and irregularity of the metacarpals and proximal phalanges are also found in patients 1 and 3. Delayed carpal bone age is detected in all three patients, but it is more severe in patients 1 and 3. No carpal ossifications are visible in patient 3 at 5 years. Brachydactyly is not apparent in all.

The major skeletal findings in the four index patients with SEMD were very similar, although severity varied significantly. The most striking features included disproportionate short stature and severe delayed ossification at the metaphyseal and epiphyseal sites. All clinical features are summarized in the clinical reports (Supplemental Materials and Methods), in Table 1, and shown in Fig. 1 and Supplemental Fig. S1.

**Table 1:** Clinical Features of the Four Index Patients With SEMD

	Patient 1	Patient 2	Patient 3	Patient 4
Ethnicity	Finnish	Korean	Korean	French/Congolese
Sex	Male	Female	Male	Male
Age (years)	4.5	4.6	3.4	Deceased at 3
Duration of pregnancy	38 + 1 weeks	40 weeks	37 + 1 weeks	37 weeks
Birth weight	2445 g (−2.3 SD)	NA	NA	NA
Birth length	42 cm (−4.5 SD)	NA	NA	NA
Head circumference at birth	34.8 cm (0 SD)	NA	NA	NA
Height	69.2 cm (−9.4 SD)	84.9 cm (−4.4 SD)	90.5 cm (−7.9 SD)	53 cm (−4 SD)
Facial dysmorphism	Mild	Mild	None	NA
Chest anomalies	Narrow thorax	Narrow thorax	Pectus excavatum	Narrow thorax
Scoliosis/lordosis	Both	Scoliosis	Scoliosis	Lordosis
Coxa vara/genu valgum	Coxa vara	NA	NA	NA
Hematological/ immunological impairments	No	No	No	No
Cognitive impairment	No	No	No	No
Hearing	Normal	Normal	Normal	Normal
Vision	Normal	Normal	Normal	NA
Dental anomalies	Prenatal hypoplasia and hypomineralization of the enamel; one extra tooth	NA	None	NA
Surgeries	None	None	Yes	None
Main complaints/other features	NA	None	Airway narrowing after general anesthesia	Abnormal sleep

SEMD = spondyloepimetaphyseal dysplasia; SD = standard deviation; NA = not available.

### *RPL13* variants underlying SEMD with severe short stature

**Fig 2:**
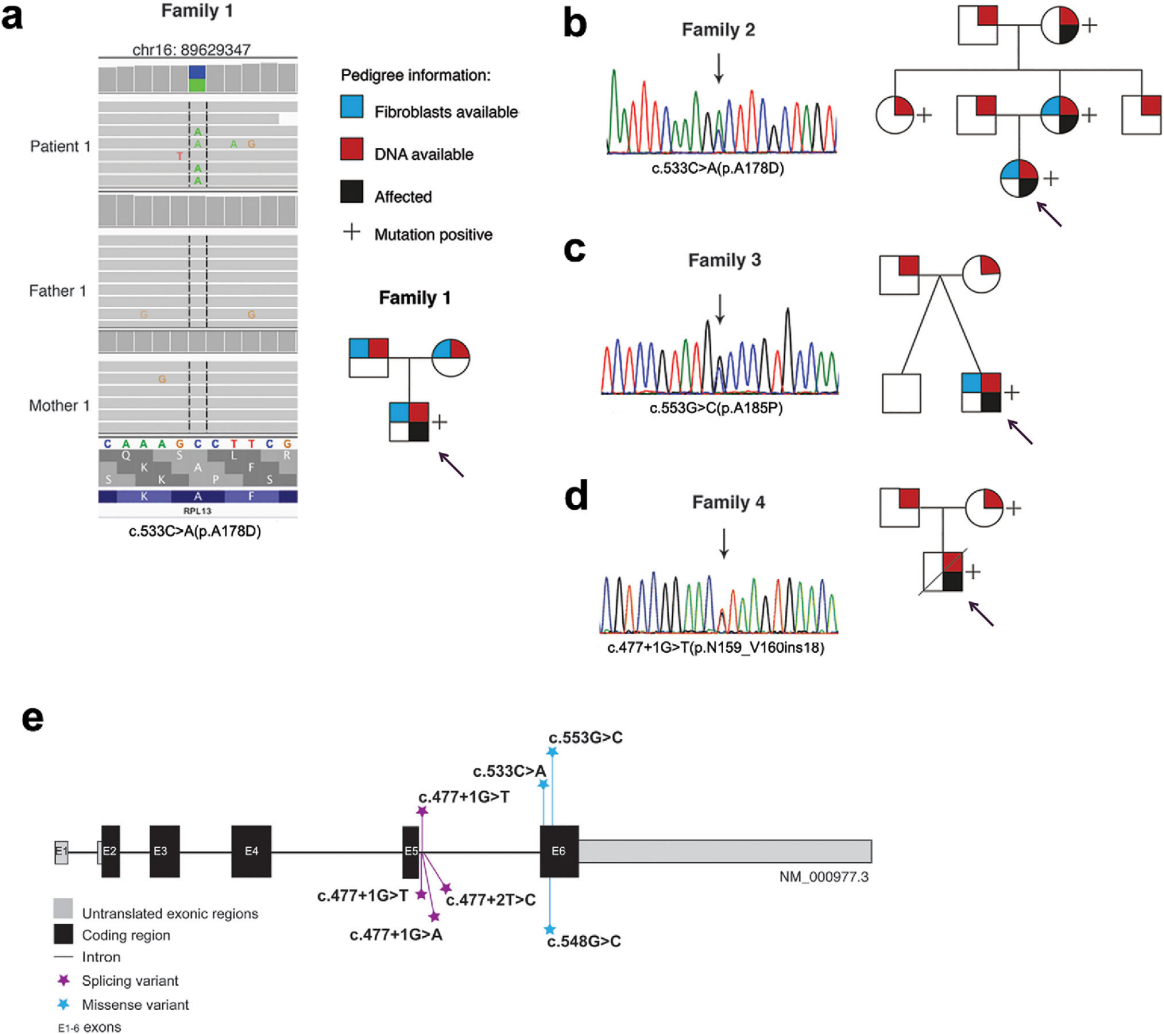
Genetic findings in the *RPL13* gene. Genetic variants and pedigrees of the four families. *RPL13* mutations were identified by WGS in family 1 (*A*), by Sanger sequencing in families 2 and 3 (*B*, *C*), and by WES followed by Sanger sequencing in family 4 (*D*). Schematic illustration of the *RPL13* gene showing all the variants identified in this report (marked above the gene) and previously reported (below the gene) (*E*).

No variant in any gene listed in the OMIM database and linked to SEMD or other osteochondrodysplasia was identified in patient 1 at the time of analysis. However, two *de novo* candidate variants were identified by WGS: (i) a heterozygous missense mutation in the *UBC* gene, NM_021009.6: c.2045G>A (p.R682K), which is also reported in one subject in the gnomAD database^(^ ^40^ ^)^ and (ii) a novel heterozygous missense mutation in the *RPL13* gene, NM_000977.3: c.533C>A (p.A178D) (Fig. 2*A*). Both mutations lie within the last exon of the respective gene. The first variant affected the gene encoding ubiquitin C, a polyubiquitin precursor. Both SIFT and Polyphen‐2 classified this SNV as likely benign. The second variant affected the gene encoding the ribosomal protein eL13. *RPL13* mutations were not linked to any disease at the time of the analysis. However, mutations in genes encoding other RPs or partaking in ribosome biogenesis had been identified in patients with hematological and skeletal impairments.^(^ ^8^, ^9^, ^16^ ^)^ This SNV is absent from the gnomAD database, but another missense variant in the same codon, p.A178V, has been described once. The variant was predicted to be deleterious by most *in silico* programs (Table 2).

**Table 2:** Description of the *RPL13* Mutations and Predictions for Their Pathogenicity

	Patient 1	Patient 2	Patient 3	Patient 4
Mutation at the DNA level	c.533C>A^a^	c.533C>A^a^	c.553G>C^a^	c.477 + 1G>T^a^
Mutation at the protein level	p.A178D; missense	p.A178D; missense	p.A185P; missense	p.N159_V160ins18; splice site
Inherited or *de novo*	*De novo*	Inherited from mother; aunt and grandmother share the same mutation	*De novo*	Inherited from mother
GERP score	4.52	4.52	3.56	4.47
CADD score (phred)	23.6	23.6	25.9	23.4
SIFT prediction	Deleterious	Deleterious	Deleterious	NA
Polyphen‐2 prediction	Benign	Benign	Probably damaging	NA
PROVEAN	Deleterious	Deleterious	Deleterious	NA
M‐CAP	Possibly pathogenic	Possibly pathogenic	Possibly pathogenic	NA

NA = not available.

^a^ Reference sequence: NM_000977.3.

WES in patient 4 led to the identification of a heterozygous genetic variant located in intron 5 of *RPL13*, c.477+1G>T (Fig. 2*D*; Table 2). This variant, recently reported also by Le Caignec and colleagues, affects the splicing mechanism leading to a longer transcript and a larger protein (p.N159_V160ins18).^(^ ^7^ ^)^ Patient 4 inherited this variant from his mother.

Considering the *RPL13* variant as the likely candidate for the disease in patients 1 and 4, we sequenced *RPL13* in patients 2 and 3 displaying the same skeletal phenotype. Sanger sequencing revealed a heterozygous *RPL13* mutation in each patient.

Patient 2 had inherited the same missense mutation c.533C>A (p.A178D) as detected in patient 1 from her mother (Fig. 2*B*; Table 2).

Patient 3 had a *de novo* heterozygous missense mutation c.553G>C (p.A185P) located 20 nucleotides downstream of the one identified in patients 1 and 2 (Fig. 2*C*; Table 2). This mutation is absent in gnomAD, but a mutation affecting the same codon, c.554C>T (p.A185V), has been reported five times in this database. All applied prediction programs predicted the p.A185P change to be deleterious (Table 2).

All the reported variants affect either the splicing donor site of intron 5 or exon 6 of *RPL13* (Fig. 2*E*).

### Incomplete penetrance and variable clinical expressivity in families 2 and 4

Our genetic analyses showed that patient 2 had inherited the missense mutation c.533C>A (p.A178D) from her mother. Interestingly, in addition to the mother, also the grandmother was found to harbor the same *RPL13* variant (Fig. 2*B*). Review of their clinical and radiological data revealed skeletal abnormalities, although remarkably milder than in the index (Supplemental Fig. S2). The mother is short (144.5 cm, −3.46 SD), has mild scoliosis, and has undergone surgery to correct pectus excavatum. She has had pain in ankles and hips since childhood and had recently sustained both spine and hip fractures. The grandmother is also short (135.8 cm, −5.6 SD), has had joint pain, especially at the knees, since adolescence, and genua valga. Radiography detected coxa vara in both the mother and grandmother (Supplemental Fig. S2), being more severe in the latter. Further, the same *RPL13* variant was also identified in the patient's aunt (157.9 cm, −0.6 SD), who does not show any radiological skeletal abnormalities (Supplemental Fig. S2).

In family 4, the clinically unaffected mother of patient 4 was found to harbor the same *RPL13* splice site variant as her child (Fig. 2D), who had severe SEMD and died during early childhood. The mother's height is 168 cm; no skeletal radiographs were available.

These findings in families 2 and 4 suggest variable disease expressivity in individuals harboring an *RPL13* mutation.

### Predicted effects of *RPL13* variants on the protein structure

**Fig 3:**
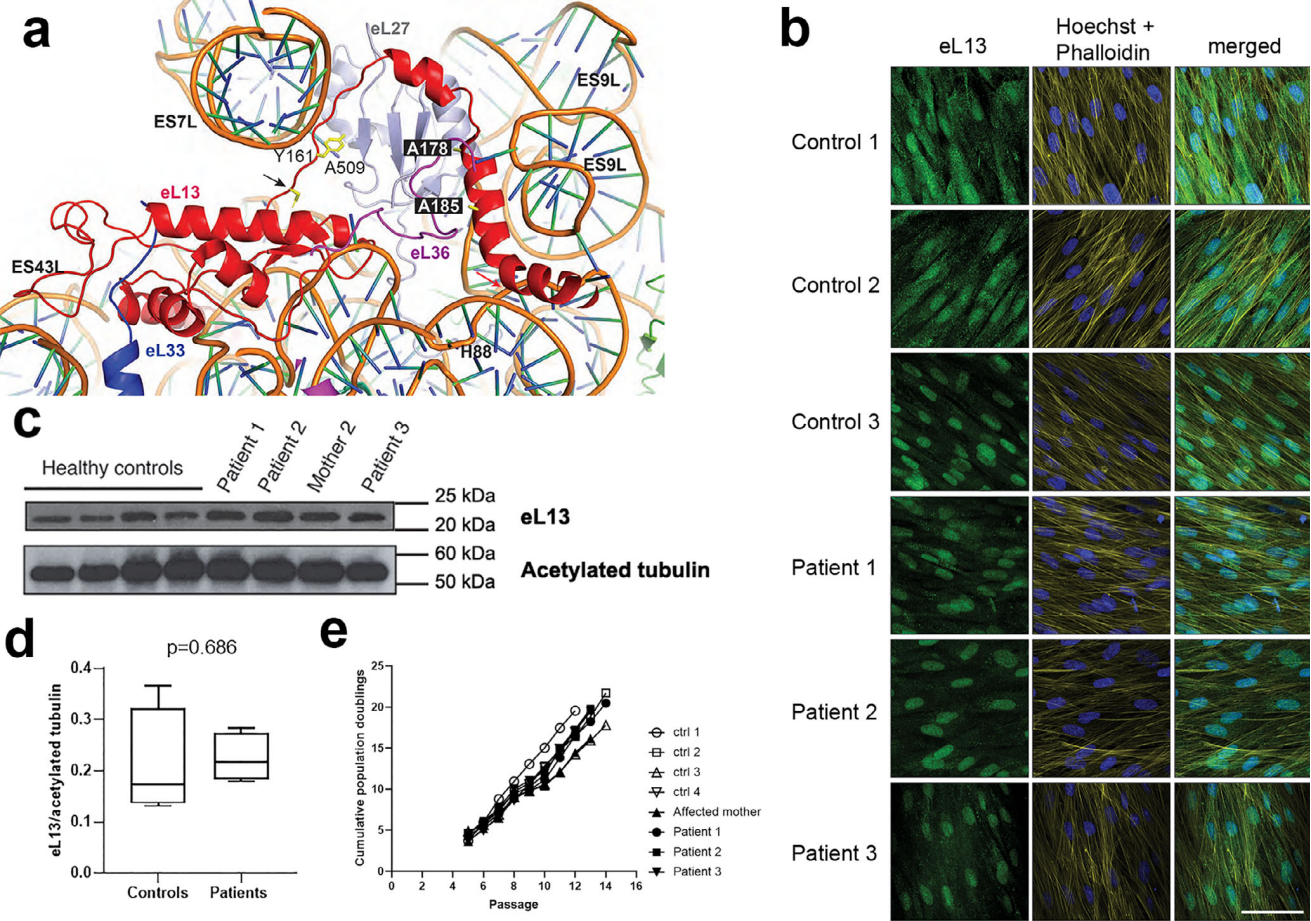
Expression of the eL13 protein in dermal fibroblasts from mutation‐positive and control subjects. (*A*) Ribbon representation of eL13 bound to the human 60S subunit (based on pdb code 4V6X). Residues in eL13 targeted by the two missense mutations are highlighted in black boxes. The starting position of the affected region caused by the intronic mutation at N159 (p.N159_V160ins18) (black arrow) and the potentially disrupted interaction between Y161 and nucleotide A509 are highlighted. The start of the frameshift deletion harbored by our zebrafish model, p.L191Afs*32, which occurs at the point where the C‐terminal α‐helix of eL13 binds to the rRNA, is also marked (red arrow). Ribosomal proteins that interact with eL13 (red) include eL33 (blue), eL36 (purple), or eL27 (gray) are shown. Figure was generated using the PyMOL molecular graphics system, version 2.0.7 (Schrödinger, LLC). (*B*) Representative immunofluorescence images of dermal fibroblasts from patients and healthy controls showing staining for eL13 (column 1), structural dyes Hoechst and Phalloidin (column 2), and merged (column 3). Scale bar = 30 μm. (*C*) Western immunoblotting (WB) demonstrated expression of the eL13 protein in dermal fibroblasts from mutation‐positive subjects at similar levels as in fibroblasts from control subjects. Acetylated tubulin was used as a loading control. (*D*) Densitometry analysis of the immunoblot confirmed the lack of significant differences in eL13 expression (mean ± SD, Student's *t* test). (*E*) Dermal fibroblasts from patients and controls showed similar growth kinetics over time in culture. Control 1 = male, age 29 years; control 2 = female age 30 years; control 3 = male age 17 years; control 4 = female age 31 years.

We set out to interpret the potential consequences of the identified mutations by examining the structure of the eL13 protein in the context of the human ribosome (pdb codes 6EK0 and 4V6X). The eL13 protein binds to the large ribosomal subunit, straddling between 28S rRNA expansion segments ES7L, ES9L, and ES43L and interacting with several ribosomal proteins including eL27, the eL36 N‐terminus, and the eL33 C‐terminus (Fig. 3*A*). The disease‐associated missense mutations A178D and A185P map to two highly conserved residues within the C‐terminal α‐helix of eL13 (high GERP score, Table 2). Introduction of a charged aspartate residue (p.A178D) is likely to destabilize the eL13 C‐terminal α‐helix and disrupt the interaction with eL27. Introduction of a proline residue (p.A185P) likely breaks the C‐terminal α‐helix, disrupting the interactions with the 28S rRNA ES9L. The third intronic mutation (p.N159_V160ins18) is predicted to result in the incorporation of 18 additional residues within an extended loop of eL13 that interacts with ES7L. We concur with Le Caignec and colleagues^(^ ^7^ ^)^ that this mutation may disrupt the specific interaction between eL13 residue Y161 and ES7L nucleotide A509. All the identified disease mutations are likely to impair the function of eL13 in the ribosome.

### Mutated eL13 is expressed in patient‐derived fibroblasts

Since our structural analysis indicated that the *RPL13* mutations are likely to affect eL13, we studied patient‐derived fibroblasts to investigate the expression and the subcellular localization of the mutated protein. Immunofluorescence staining demonstrated presence of eL13 in patient‐derived cells (Fig. 3*B*), with distribution in both the nucleus and the perinuclear area, the latter representing the location of the endoplasmic reticulum (ER). In most samples, eL13 located predominantly to the nucleus. WB revealed no significant differences in protein expression between the control group and the patient group (Fig. 3*C*, *D*). These analyses suggest that the mutated protein is not degraded. Patient‐derived fibroblasts displayed similar growth kinetics over time as fibroblasts from controls (Fig. 3*E*).

### eL13 subcellular localization and colocalization with other RPs

**Fig 4:**
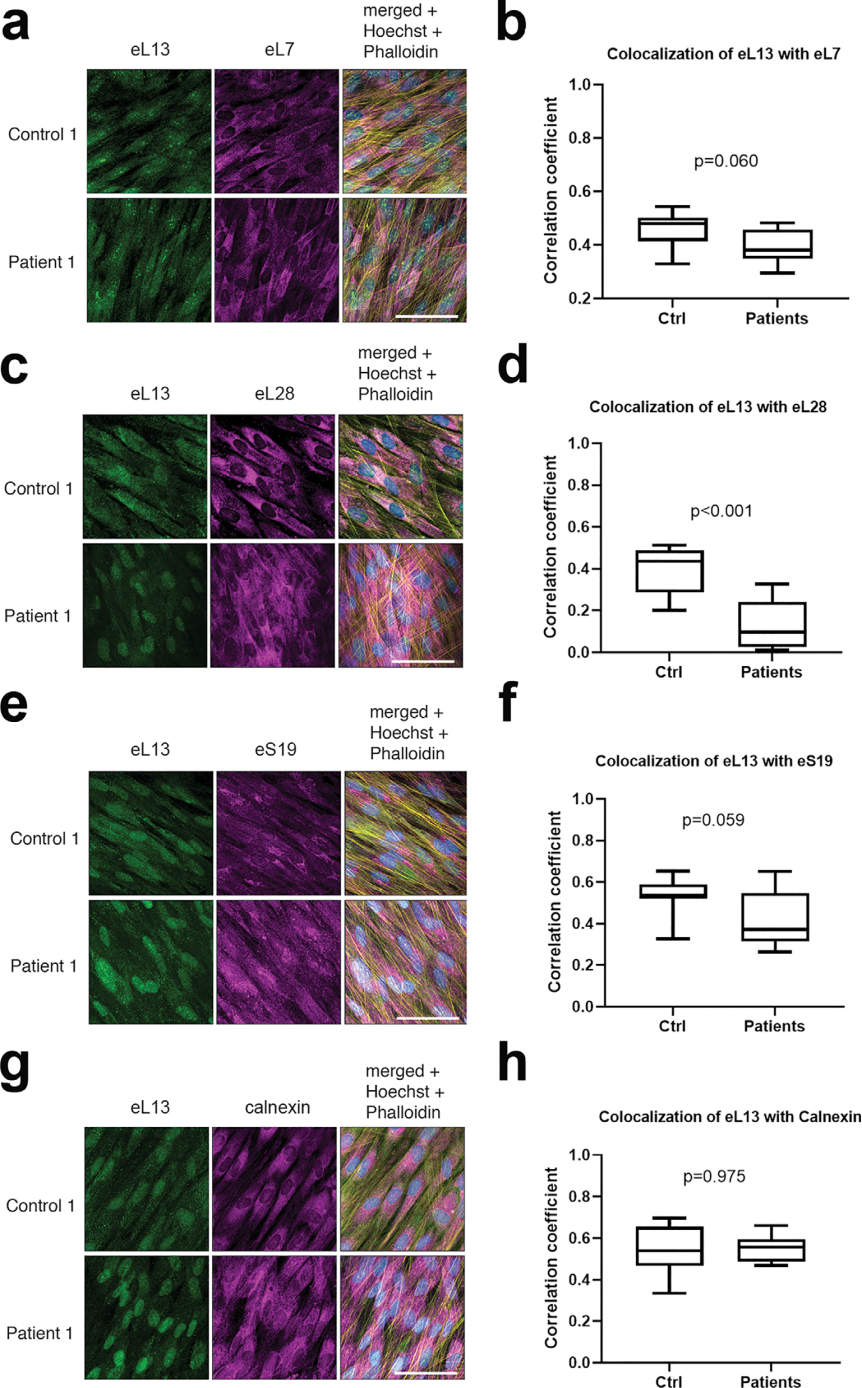
Comparison of colocalization of eL13 with other ribosomal proteins and with ER. Representative immunofluorescence images of dermal fibroblasts from patient 1 and one healthy control showing co‐staining of eL13 with eL7 (*A*), eL28 (*C*), eS19 (*E*), and with calnexin as a marker of ER (*G*). Scale bars = 30 μm. 3D colocalization of mutant and WT eL13 with the selected ribosomal markers was analyzed from entire z‐stacks of three samples from patients 1 to 3 (*n* = 3) and each healthy control (*n* = 3) using the colocalization test plugin of ImageJ Fiji and is expressed as correlation coefficient (*B*, *D*, *F*, *H*). Box plots with median and range; *p* values from Mann–Whitney *U* test.

Co‐stainings of eL13 with ribosomal protein eL7 and eL28 were conducted to examine assembly of eL13 to large ribosomal subunit (60S); both suggested a similar subcellular location in control and patient cells (Fig. 4*A*, *C*). Colocalization analysis showed a medium agreement in localization between eL13 and eL7 in control (0.48, 0.33–0.54) and patient cells (0.38, 0.30–0.48) with no significant difference (*p* = 0.060) (Fig. 4*B*). However, eL28 demonstrated low colocalization with eL13, being significantly lower in patient (0.1, 0.01–0.33) than control cells (0.44, 0.20–0.51, *p* < 0.001) (Fig. 4*D*). Co‐staining with ribosomal protein S19 was done to investigate if the 60S' harboring mutated eL13 could colocalize with the smaller ribosomal subunit (40S) (Fig. 4*E*). A median level colocalization was observed in both control (0.54, 0.33–0.65) and patient cells (0.37, 0.26–0.65) without significant difference (*p* = 0.059) (Fig. 4*F*). Calnexin staining was performed to measure ribosomes' localization to ER; co‐staining with eL13 showed similar localization in patient (0.56, 0.47–0.66) and control cells (0.54, 0.33–0.70, *p* = 0.975) (Fig. 4*G*, *H*).

### Increased 60S:80 ratio and impaired global translation in mutation‐positive subjects

**Fig 5:**
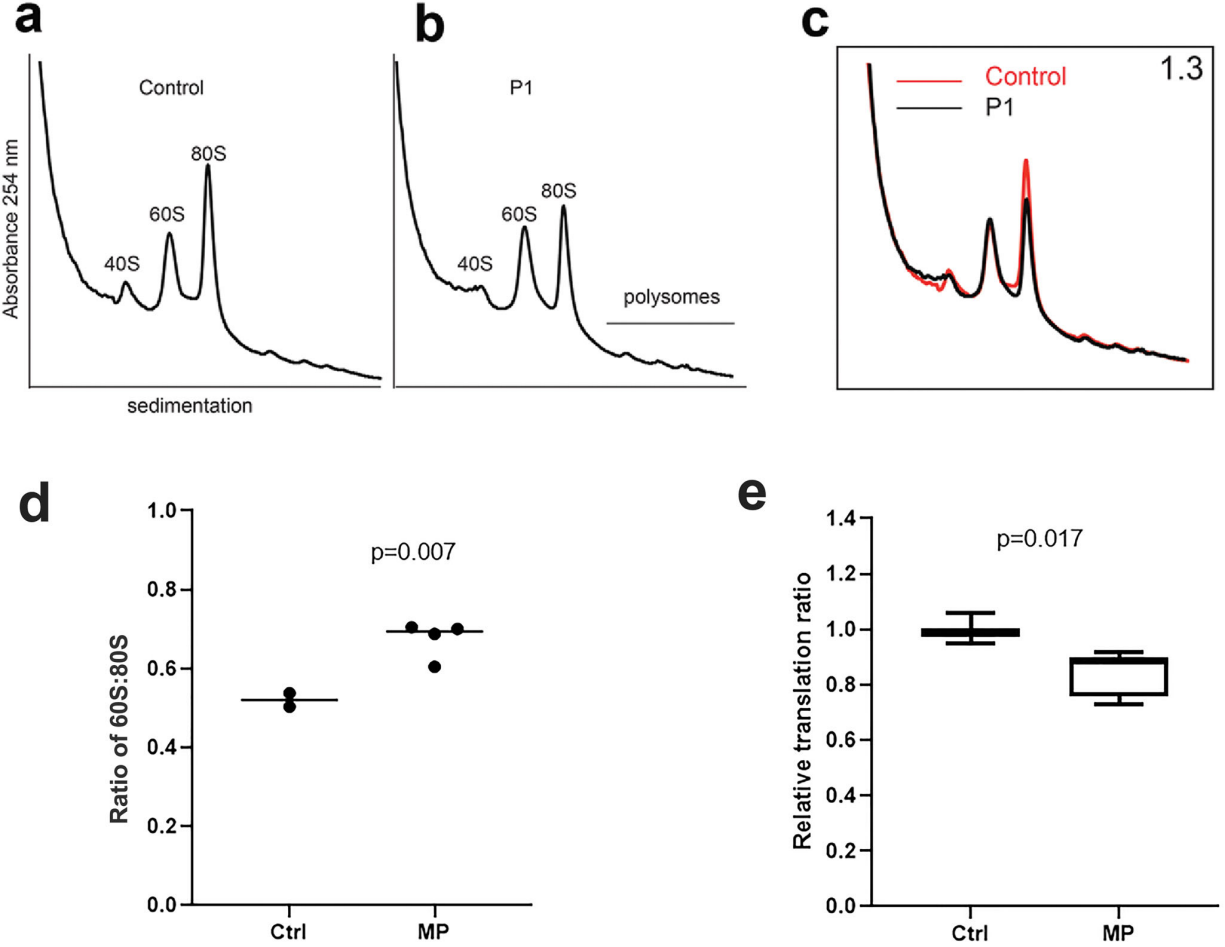
Sucrose profiles and global translation in patients 1, 2, and 3. Comparison of ribosomal fractions from fibroblasts of healthy control parent (*A*) and affected patient 1 (P1) heterozygous for the p.A178D *RPL13* mutation (*B*). (*C*) Overlay of sucrose gradient profiles derived from healthy control parent (red line) and affected individual P1 (black line). Ribosomal subunit ratio (60S:80S) is indicated in the top right corner. (*D*) Box plot showing the results of the 60S:80S from fibroblasts of healthy controls (parents of patient 1) and mutation‐positive (MP) subjects (patients 1 to 3 and mother of patient 2), each of whom is heterozygous for an *RPL13* mutation. An increase median value for the 60S:80S ratio is observed in the MP group. The *p* value from one‐sample *t* test comparing mutation‐positive group against the median of the control samples. (*E*) Relative translation ratio from OP‐puro translation assay. Global protein rate is reduced in the MP subjects (patients 1 to 3 and mother of patient 2) compared with controls (*n* = 2). Data are presented as mean and interquartile range. Experiments were performed twice, in duplicate or triplicate/donor/experiment, and analyzed using Mann–Whitney *U* test.

Compared with control fibroblasts derived from the parents of patient 1 who lack mutations in eL13, sucrose gradient sedimentation of cell extracts from fibroblasts derived from mutation‐positive subjects showed a significant increase in the ratio of 60S subunits to 80S ribosomes (*p* = 0.007) (Fig. 5*A*–*D*). Furthermore, compared with controls, global rates of protein synthesis were significantly reduced in fibroblasts from mutation‐positive subjects as assessed by *in vivo* incorporation of O‐propargyl‐puromycin (*p* = 0.017) (Fig. 5*E*). Taken together, these data indicate that disease mutations in eL13 impair global translation.

### *rpl13*^L191Afs^/^L191Afs^ zebrafish features skeletal abnormalities

**Fig 6:**
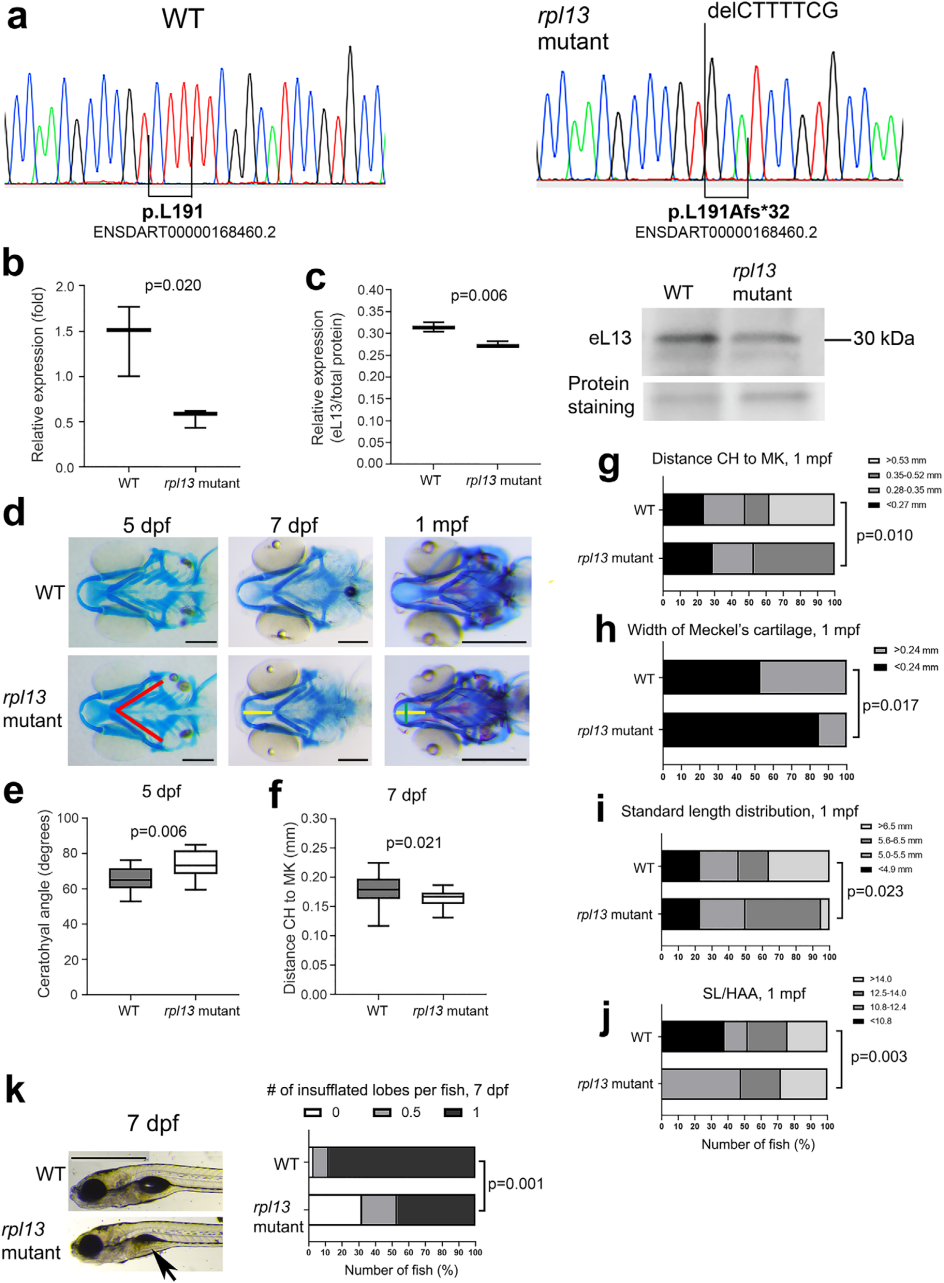
Evaluation of the *rpl13* ^*L191Afs*^/^*L191Afs*^ zebrafish. (*A*) Sanger sequencing confirmed the genotype of the homozygous *rpl13* ^*L191Afs/L191Afs*^ (*rpl13* mutant) fish. (*B*) qPCR performed on RNA extracted from three WT and three *rpl13* ^*L191Afs*^ */* ^*L191Afs*^ reveals a partial activation of nonsense‐mediated mRNA decay in mutant zebrafish compared with WT. The *p* value from Student's *t* test. (*C*) WB performed on protein extracts from three WT and three *rpl13* ^*L191Afs*^ */* ^*L191Afs*^ fish reveals reduced expression of eL13 in mutant fish compared with WT. The *p* value from Student's *t* test. (*D*) Alcian blue staining of control (WT) and *rpl13* ^*L191Af*^ */* ^*L191Afs*^ zebrafish at 5 dpf, 7 dpf, and 1 mpf. Compared with WT, cartilage deformities in the head of the *rpl13* ^*L191Afs/L191Afs*^ are evident. Red lines indicate the ceratohyal (CH) angle, yellow line indicates the distance between the tip of CH and the tip of Meckel's (MK) cartilages, and green line indicates the MK width. Scale bar at 5 and 7 dpf = 200 μm; scale bar at 1 mpf = 500 μm. (*E*) At 5 dpf, *rpl13* ^*L191Af/L191Afs*^ show a significant increase of the CH angle compared with WT and (*F*) at 7 dpf a significant reduction in the distance between the CH and MK cartilages is evident in mutants compared with WT. The *p* values from Student's *t* test. (*G*) At 1 mpf, the frequency of fish with short distance (<0.53 mm) between the CH and MK cartilages and (*H*) the frequency of fish with narrower MK cartilage were significantly higher in mutants compared with WT. The *p* values from chi‐square test. (*I*) Standard length (SL) distribution is significantly different in WT compared with *rpl13* mutants at 1 mpf, with a higher number of long fish (>6.5 mm) in the WT population compared with mutants. The *p* value from chi‐square test. (*J*) A higher frequency of fish with high SL/HAA (height at anterior margin of anal fin) ratio is found in the mutant group compared with WT group. Morphometric parameters (panels G‐J) were categorized by global quartiles, presented as number of fish (%) in each category, and frequencies analyzed by chi‐square test. (*K*) Swim bladder inflation in control (WT) and *rpl13* ^*L191Af/L191Afs*^ zebrafish at 7 dpf. Arrows indicate a partially inflated lobe in mutants compared with WT. Scale bar = 1 mm. Quantitative analysis shows a significant delay in the swim bladder inflation of the first lobe in *rpl13* ^*L191Af/L191Afs*^ compared with WT. The *p* values from crosstabs with chi‐square test for frequencies.

Homozygous *rpl13* mutant fish harbored a seven‐nucleotide deletion, c.571_577delCTTTTCG, responsible for a frameshift predicted to alter the amino acid sequence and to elongate the C‐terminal end of the protein by 11 amino acids, L191Afs*32 (Fig. 6*A*). The mutation occurs at the site where the C‐terminal α‐helix of eL13 binds to the rRNA (Fig. 3*A*). The terms “*rpl13* mutant fish” and “*rpl13* ^*L191Afs*^ */* ^*L191Afs*^ *”* are used for the fish harboring the homozygous p.L191Afs*32 change.

qPCR analysis revealed a partial activation of nonsense‐mediated mRNA decay, with 62% reduction of *rpl13* expression in mutant compared with WT (*p* = 0.02) (Fig. 6*B*). The mutant eL13 protein is translated in *rpl13* ^*L191Afs*^ */* ^*L191Afs*^ fish but with a 14% reduced level compared with WT (*p* = 0.006) (Fig. 6*C*).

Mendelian ratios at 5 dpf, 7 dpf, and 1 mpf indicate that there is no significantly reduced viability of homozygous *rpl13* mutant zebrafish at these stages. Homozygous *rpl13* mutant fish presented with cranial cartilage deformities. At 5 dpf, the CH was significantly wider in mutant embryos (Fig. 6*D‐E*) and at 7 dpf the distance between the tip of CH and the tip of MK was reduced (Fig. 6*D, F*). At 1 mpf, homozygous *rpl13* mutants showed both a reduced distance between the tip of CH and the tip of MK (Fig. 6*G*) and a narrower MK compared with WT (Fig. 6*H*), indicating craniofacial developmental defects. Compared with WT fish, a significant difference in standard length distribution (Fig. 6*I*), a significant increase in the SL/HAA ratio (Fig. 6*J*), and a normal SOL/HE ratio were detected in *rpl13* ^*L191Afs*^ */* ^*L191Afs*^ at 1 mpf, thus supporting the presence of body disproportions. Finally, *rpl13* ^*L191Afs*^ */* ^*L191Afs*^ embryos showed a significant delay in swim bladder inflation at both 5 and 7 dpf compared with WT (Fig. 6*K*). This feature was rescued at 1 mpf.

## Discussion

In the present study, we provide significant new data on a novel ribosomopathy with major skeletal involvement and absent extraskeletal manifestations. Our study confirms an association between monoallelic variants in *RPL13* and SEMD in four unrelated families. Moreover, we also describe novel features in SEMD‐RPL13 type, including incomplete penetrance and variable clinical expressivity within families harboring the same mutation. Cellular defects in these individuals include a reduction in 80S ribosomes and attenuated global translation. Furthermore, we modeled the human condition in zebrafish.

Only one study has previously reported on *RPL13* mutations in four patients with skeletal dysplasia and negative family history.^(^ ^7^ ^)^ We here report on four additional families with altogether eight mutation‐positive subjects. We identified two novel missense *RPL13* mutations and one previously reported splicing variant. The two missense mutations, p.A178D and p.A185P, affect two residues that map to the C‐terminal α‐helix of eL13. At the DNA level, both variants affect exon 6. The third mutation, altering the splice donor site in intron 5, leads to partial intron retention, thus generating an elongated protein (p.N159_V160ins18).^(^ ^7^ ^)^ This mutation has now been reported twice and a different mutation affecting the same nucleotide has been reported once before.^(^ ^7^ ^)^ Interestingly, all the currently known *RPL13* mutations associated with SEMD cluster within the same region, suggesting that this region is critical for eL13 function, especially in skeletal tissues. According to our *in silico* predictions, the identified mutations are likely to disrupt the interaction of eL13 with either eL27 or the 28S rRNA, thus leading to ribosomal dysfunction. Previous studies have shown that haploinsufficiency of genes encoding other RPs or some ribosomal components lead to ribosomopathies with skeletal involvement.^(^ ^8^, ^9^ ^)^ For instance, monoallelic mutations in 19 different RP genes are responsible for Diamond‐Blackfan anemia,^(^ ^41^ ^)^ characterized by congenital erythroid aplasia but also skeletal defects in 40% to 62% of cases.^(^ ^42^, ^43^ ^)^

Our four index patients, all feature a similar phenotype, characterized by short stature and severe skeletal impairments with vertebral abnormalities and delayed ossification at the metaphyseal and epiphyseal sites. Although patient 2 has a milder phenotype, comparable to the recently reported SEMD patients with *RPL13* mutations,^(^ ^7^ ^)^ patients 1, 3, and 4 feature more severe skeletal changes and are likely to represent the severest end of the disease spectrum. In contrast to other patients with ribosomopathies, our patients did not present any remarkable extraskeletal defects such as hematological or immunological manifestations, which is in line with the recent report by Le Caignec and colleagues. Based on the clinical phenotypes with bone and cartilage tissue specificity, it can be speculated that functional eL13 is of special importance in the growth plate and sites of endochondral ossification. Production of the unique ECM vital for normal growth plate development poses high translational demands to the chondrocytes of the growth plate,^(^ ^9^, ^44^ ^)^ possibly making them more sensitive to changes in protein synthesis than other cell types. This hypothesis is supported by the skeletal phenotypes in ribosomopathies,^(^ ^8^, ^9^ ^)^ and recent findings indicating that genes involved in ribosome biogenesis are upregulated in human articular chondrocytes.^(^ ^45^ ^)^ Moreover, some RPs are also likely to have tissue‐specific functions, thus leading to a unique spectrum of manifestations in each ribosomopathy.^(^ ^46^ ^)^

This is the first report describing extreme variability in phenotypic expression in individuals with disease‐causing *RPL13* variants. In family 2, the index patient is the most severely affected, followed by her grandmother and mother, respectively. On the other hand, the aunt does not present any skeletal impairments despite harboring the same heterozygous *RPL13* mutation. Similarly, the mutation‐positive mother in family 4 has no clinical manifestations. Increased expression from the wild‐type allele or even monoallelic expression or presence of protective genetic modifiers might impede the disease development in healthy or mildly affected subjects harboring *RPL13* mutations. Since RPs have multiple pseudogenes dispersed throughout the genome,^(47^ ^)^ a partially functional *RPL13* pseudogene could also compensate for the activity of the impaired “parental gene”. Further, lifestyle factors or even chance could impact disease expression in subjects harboring the mutation. Moreover, it is possible that epimetaphyseal changes ameliorate with increasing age and skeletal maturation. Such observation has been reported in patients with some other osteochondrodysplasias, including spondylometaphyseal dysplasia with “corner fractures” caused by mutations in the fibronectin gene and metaphyseal anadysplasia caused by mutations in metalloproteinases.^(^ ^48^, ^49^, ^50^, ^51^ ^)^ Interestingly, incomplete penetrance and variable expression among patients with identical mutations is a common feature in ribosomopathies,^(^ ^8^ ^)^ but the underlying mechanisms still remain largely unknown.

When investigating consequences of the *RPL13* mutations in dermal fibroblasts, we identified a significant decrease in colocalization of eL13 with eL28 in patient‐derived cells. This might indicate altered assembly and/or function of the 80S, supported by increased 60S:80S ratio and attenuated global translation rates detected in subjects harboring *RPL13* mutations. Because an increased 60S:80S ratio was also detected in a patient with an *RPL13* splice site mutation,^(^ ^7^ ^)^ we can speculate that both missense and splicing mutations lead to similar outcome. Nevertheless, further studies are needed to confirm this hypothesis. The similar growth kinetic of patient‐ and control‐derived fibroblasts despite the patients being younger (4 to 12 years) than controls (17 to 31 years) could be an indirect indicator of decreased proliferative capacity due to *RPL13* mutations, based on the well‐established age‐related decline in cell proliferation^(^ ^52^, ^53^, ^54^ ^)^ and the well‐known importance of ribosome biogenesis in general cell growth and cell‐cycle regulation.^(^ ^9^ ^)^

As a further approach to elucidate disease mechanisms, we established an *rpl13* mutant zebrafish model harboring a frameshift deletion. The *rpl13* ^L191Afs^/^L191Afs^ fish partly recapitulated the phenotype of patients with *RPL13* mutations by featuring cartilage deformities both at embryonic and juvenile stage of development. *rpl13* mutants at 1 mpf also show a prevalence of short standard length and an increased SL/HAA ratio compared with WT fish, thus indicating some degree of body disproportion, which is a common feature in SEMD‐RPL13 type. In addition, the delay in swim bladder inflation in 5 and 7 dpf homozygous *rpl13* ^L191Afs^/^L191Afs^ larvae suggests possible general developmental delay. Although our zebrafish mutant does not fully reproduce the patients' molecular defects and mutated *rpl13* is expressed at both transcript and protein levels, it is likely that the introduced mutation impairs the structure and function of eL13 by disrupting the binding of the C‐terminal α‐helix to rRNA. Therefore, our zebrafish model represents a proof‐of‐principle for the relevant role of eL13 in skeletal development and a valuable tool for further studies at later stages.

Collectively, our findings delineate and expand the SEMD‐RPL13 type by reporting novel *RPL13* mutations in subjects with severe skeletal manifestations and by describing large variability in disease severity even within families. Moreover, our *in vitro* experiments provide direct evidence for impaired 60S:80S ratio and attenuated global translation in cells from mutation‐positive subjects. Finally, we have established an *rpl13* ^L191Afs^/^L191Afs^ zebrafish model displaying cartilage deformities partly recapitulating the human disease, which provides a model for further studies and deeper understanding of the molecular basis of the disease that could eventually be used for drug testing as well.

Despite a clear association between *RPL13* variants and SEMD, our findings from colocalization studies need to be validated by other methods that allow investigation of protein–protein interactions. Moreover, additional samples and replicates should be analyzed to confirm our results from sucrose profiling. Future studies should explore the molecular mechanisms leading to ribosome dysfunction and the consequent severe, early manifesting skeletal features in our patients and elucidate factors partially or totally preventing disease manifestation in some subjects. Additionally, a characterization of our zebrafish model at an adult stage will potentially further highlight the features common to patients with SEMD‐RPL13 type, such as reduced growth.

Our novel findings will be important for genetic counseling in families with *RPL13* mutations and for screening undiagnosed SEMD patients.

## Disclosures

All authors state that they have no conflicts of interest.

### Peer Review

The peer review history for this article is available at https://publons.com/publon/10.1002/jbmr.4177.

## Supporting information


**Supplemental Fig. S1.** Patients’ radiographs of the thorax and spine.
**Supplemental Fig. S2.** Radiographs of the mutation‐positive members in family 2.
**Supplemental Table S1.** Antibodies and Immunocytochemistry Reagents
