# Supplementary material for: Novel RPL13 Variants and Variable Clinical Expressivity in a Human Ribosomopathy With Spondyloepimetaphyseal Dysplasia
Source: J Bone Miner Res. 2020 Oct 13;36(2):283–97. doi: 10.1002/jbmr.4177 (PMC7988564; doi:10.1002/jbmr.4177)
Supplement: Supplementary file 1 — Supplemental Fig. S1. Patients’ radiographs of the thorax and spine. Supplemental Fig. S2. Radiographs of the mutation‐positive members in family 2. Supplemental Table S1. Antibodies and Immunocytochemistry Reagents [file JBMR-36-283-s001.pdf]

Supplemental Materials and Methods

Novel *RPL13* variants and variable clinical expressivity in a human ribosomopathy with spondyloepimetaphyseal dysplasia

Costantini et al.

1. Clinical reports

In Family 1, the index patient (patient 1), presently a 4.5-year-old boy, was evaluated for disproportionate short stature and severe skeletal impairments (Figs. 1 and 2). He is the only child of healthy unrelated Finnish parents. His growth retardation was noted already during pregnancy (Supplemental Table S1). Karyotype, plasma amino acids, urine organic acids, glycosaminoglycans and oligosaccharides, as well as heart and abdominal ultrasounds were all normal. At 6 months, radiographs of the spine and long bones indicated skeletal dysplasia: he had abnormal pelvis, bilateral coxa vara, short long bones with broad and flared metaphyseal areas and abnormal epiphyses. He walked independently by 2.1 years and at 3 years bracing treatment was initiated for early-onset scoliosis. At 4.5 years the patient has severe disproportionate short stature (height -9.4 SD) with large head relative to his small and slim body (Fig. 1). He has mild facial coarseness, low nasal bridge, and hypoplastic primary teeth with hypomineralization. Hearing, vision and mental development are normal. Total blood count, immunoglobulins, infection rate, and kidney and liver functions are also normal.

In Family 2, the presently 4.6-year-old girl (patient 2), was clinically investigated for short stature and spinal deformity. She is the only child of healthy unrelated Korean parents. She started walking independently at 16 months. At 2.6 years she was hospitalized due to pneumonia. A skeletal dysplasia was suspected, and she was referred to a tertiary pediatric center where scoliosis and coxa vara were detected (Fig. 1; Supplemental Figure S1) but no laboratory abnormalities were noted. Her height was -4.4 SD. Radiographs showed abnormal skeletal changes in the pelvis and tubular bones; hand radiographs showed delayed carpal ossification. She also had a short and abnormal thorax, scoliosis and spinal irregularities. Her liver, spleen and kidneys were of normal size and heart function was normal. The total blood

count, renal function, liver enzymes, thyroid hormones and serum IGF-1 and IGFBP-3 were normal.

Family 3 is of Korean origin and the index patient (patient 3) was investigated at age 3.4 years because of short stature, protruded abdomen, and spine curvature. He was one of dizygotic twins, the twin sister being healthy, conceived by *in vitro* fertilization between the healthy parents; there was no parental consanguinity. At age 4 months he had severe bronchiolitis requiring ventilator care. Motor development was delayed with independent walking at 20 months. He showed marked bilateral genu varum, lumbar lordosis, protruded abdomen, and pectus excavatum. Ligamentous laxity (Beighton score 9) was noted. Skeletal survey further showed scoliosis, spinal changes, and abnormalities in his chest and lower limbs, with most striking abnormalities at the metaphyses (Fig. 1; Supplemental Figure S1); scoliosis necessitated spinal fusion at 6.4 years. At 9.5 years his height was 90.5 cm (-7.9 SD). He underwent femoral osteotomy to correct progressive coxa vara. Postoperatively he developed progressive dyspnea and was found to have grade III subglottic stenosis, which persisted despite several trials of dilatation.

In Family 4, the index patient (patient 4) is the third child of healthy unrelated parents. The mother originates from France and Belgium and the father from Congo. Polyhydramnios was noted at 30 weeks of gestation. He was first investigated for respiratory distress related to narrow thorax. Sleep study was abnormal with numerous obstructive episodes together with bradycardia. By 2 months he developed disproportionate short stature (-4.0 SD). Karyotype, plasma amino acids, urine organic acids, as well as heart and abdominal ultrasounds were all normal. Radiographs of long bones (Fig. 1) and spine (Supplemental Figure S1) showed metaphyseal flaring and epiphyseal delay, short ribs and severe spinal anomalies with major platyspondyly. At 14 months of age, severe growth retardation persisted (-6.0 SD). He started walking independently at 22 months of age. Narrow thorax, lumbar lordosis, and protruding

abdomen were observed. Audiometry and cognitive development were normal. He died at 3 years of age due to respiratory insufficiency.

2. Supplemental figures

Supplemental Figure S1. Patients' radiographs of the thorax and spine. Thorax and spine radiographs of patient 1 (a, b), patient 2 (c, d), patient 3 (e, f) and patient 4 (g, h). The thorax appears short and broad with cupped and flared anterior ends of the ribs. The vertebral bodies show ventral biconvexity and dorsal constriction. Thoracolumbar scoliosis is evident in patients 2 and 3.

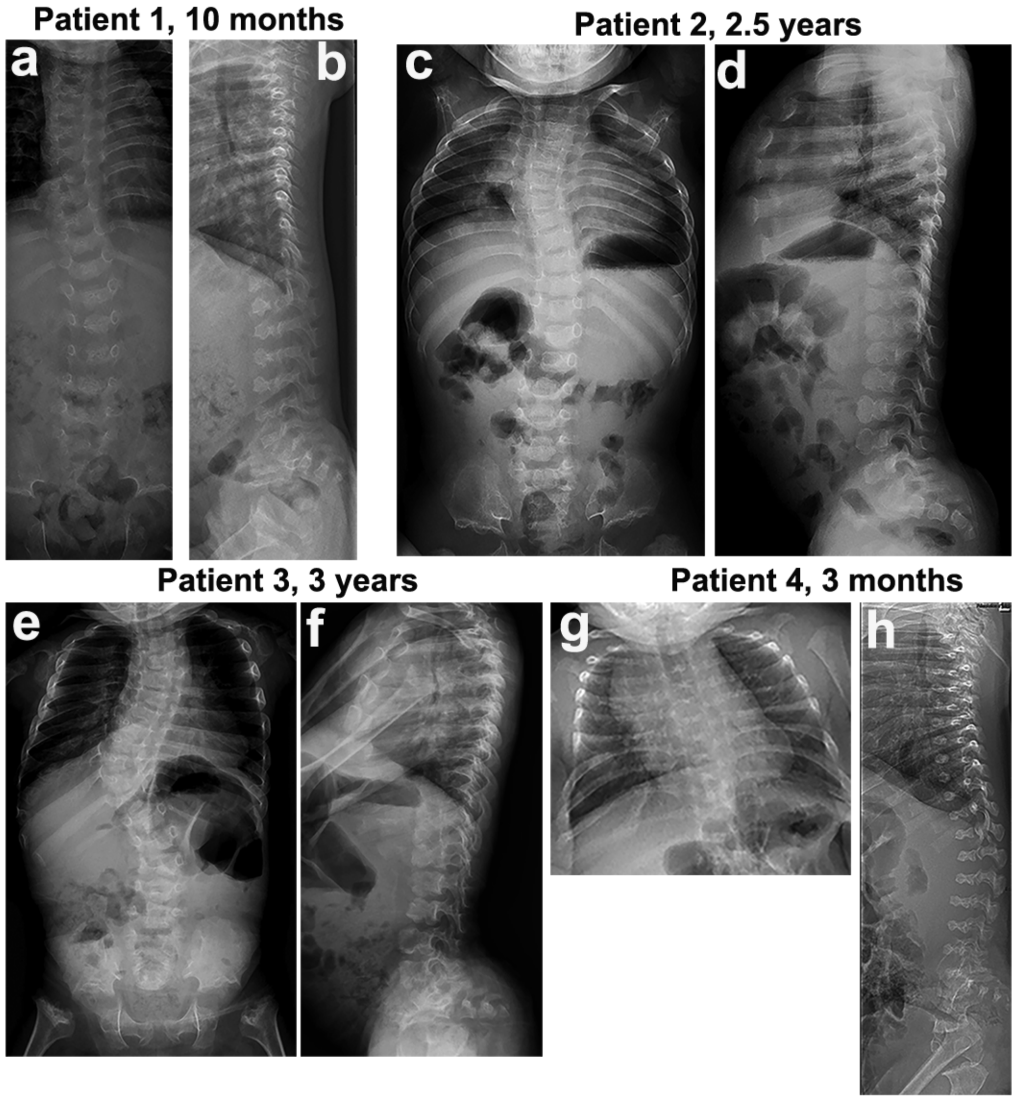

Supplemental Figure S2. Radiographs of the mutation positive members in Family 2. In Family 2 only the index patient (Fig. 1d) showed severe skeletal dysplasia while the other mutation-positive family members show mild to normal radiological findings. Radiographs of the mother at 23 years (Left panel), the maternal grandmother at 49 years (Central panel) and the aunt at 25 years (Right panel). The mother has mild scoliosis (a-c), significant endplate irregularity in the thoracic spine and short ribs. The grandmother has reduced vertebral height and abnormal vertebral shape with depressed middle parts (g-i). Both the mother (e, f) and the grandmother (k, l) have coxa vara deformity and significant degenerative changes at the proximal hip and to lesser degree in the knees. The hand radiographs (d, j) do not show major abnormalities apart from degenerative changes in the wrist in the grandmother (j). No abnormal skeletal features are detected in the aunt (m-r).

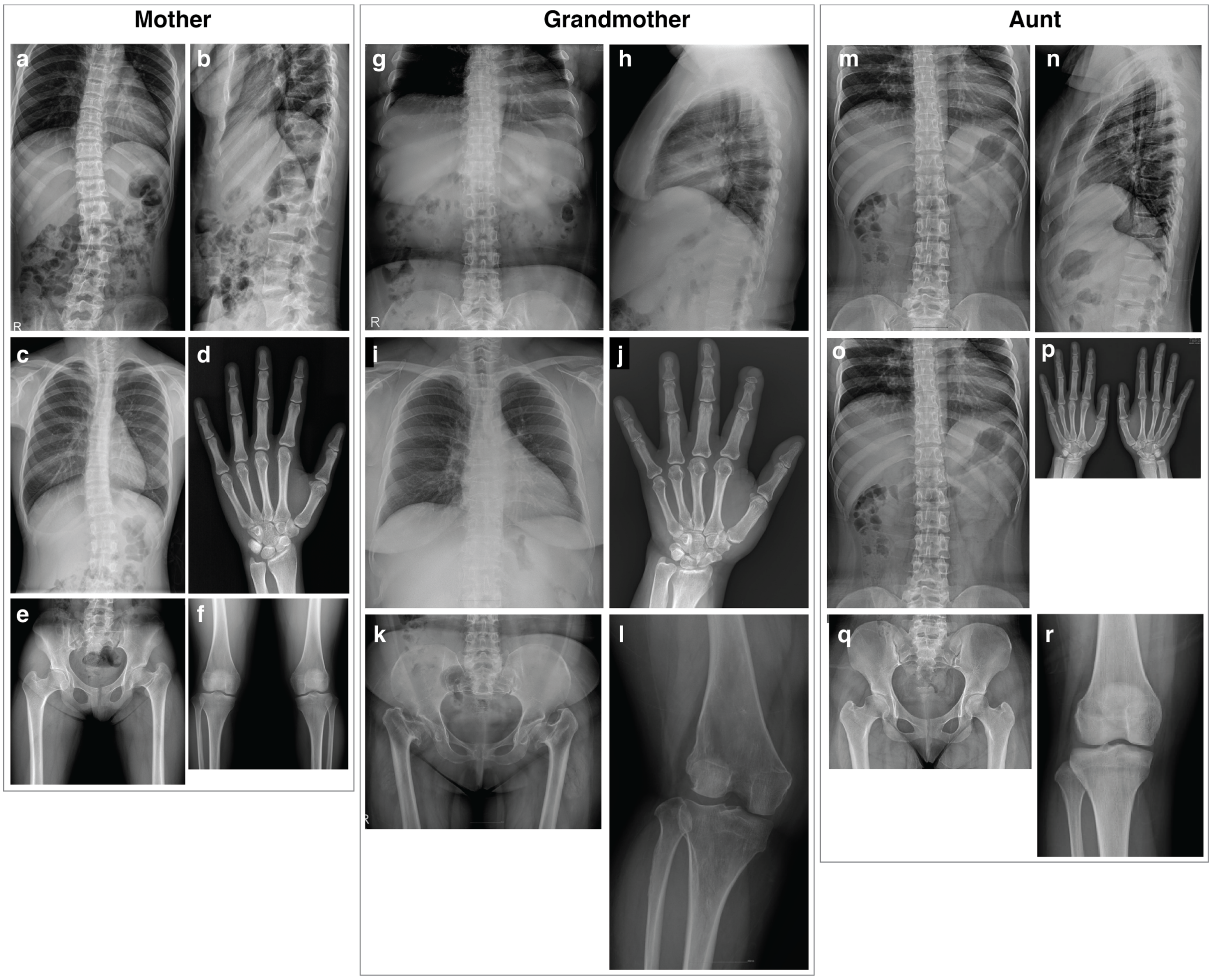

3. Supplemental notes on the methods

Whole-genome and whole-exome sequencing

For whole-genome sequencing, genomic DNA was extracted from blood according to standard procedures. Library preparation was carried out at the Science for Life Laboratory (SciLifeLab) using the Illumina TruSeq PCR-Free method. Pair-end sequencing (2 x 150 base-pair) was performed on the Illumina HiSeq X instrument at an average autosomal coverage of 30X at the same facility. Read alignment, quality control, variant calling and variant annotation were performed using our previously described in-house pipeline[1,2].

Exome capture was carried out at the genomic platform of the IMAGINE Institute (Paris, France) with the SureSelect Human All Exon kit (Agilent Technologies). Agilent SureSelect Human All Exon (V4) libraries were prepared from 3 μg of genomic DNA sheared with Ultrasonicator (Covaris) as recommended by the manufacturer. Barcoded exome libraries were pooled and sequenced using HiSep2500 (Illumina) generating paired-end reads. Read alignment, quality control, variant calling and variant annotation were performed as previously described[3].

Dermal fibroblasts culture

For isolation of patient- and control-derived dermal fibroblasts, skin biopsies were collected and transported in sterile complete culture medium (DMEM supplemented with 20% fetal bovine serum (FBS) and antibiotics (50 IU/ml penicillin, 50 μg/ml streptomycin). The biopsies were minced into smaller fragments in sterile phosphate buffered saline (PBS), samples were treated with 1000 U/ml Collagenase Type II (Clostridium histolyticum, Gibco) in Mg- and Ca-free PBS for 2h at 37 °C. Following inactivation of collagenase with ice-cold culture media and vigorous vortexing, tissue lysates were centrifuged at 150 x g for 10 min at 4 °C. Cell pellets were re-suspended in complete media, seeded in tissue culture flasks and incubated at 37 °C in a 5% CO_2 humidified incubator. Non-adherent cells were discarded after

2-3 days, and after 1-2 weeks cells were trypsinized for further expansion. Cells were expanded by seeding at 7 000-10 000 cells/cm^2 in high glucose DMEM (Thermo Scientific Cat. #11960044) supplemented with 1% GlutaMAX™ (Thermo Scientific Cat. #35050038) and antibiotics. For the first 3-4 passages, 20% FBS was used, thereafter 15%. Media was changed every 3-4 days, and cells were splitted upon 80% confluence. Cells of passage 3-6 were used for characterization assays. For monitoring the *in vitro* proliferative capacity, fibroblasts were expanded under same conditions up to passage 15 and population doublings (PDs) at each passage was calculated as *log N/log 2*, where *N* is the number of cells yielded at trypsinization divided by the number of cells seeded. Growth kinetics through several passages was expressed by calculating cumulative PDs.

Western immunoblotting

Briefly, denatured protein samples were separated on 10% SDS-Page gel followed by wet transfer to a PVDF membrane. After blocking (5% non-fat milk in 1xTBST at room temperature (RT) for 1-2 hours), membrane was incubated with primary antibody in 5% non-fat milk-TBST at 4°C overnight (o/n). Membrane was washed 3 x 15 minutes in 1xTBST before incubation with secondary antibody in blocking solution for 1h at RT. After washing 3 x 15 minutes in 1xTBST, membrane was incubated for 3-5 minutes in Bio-Rad's Clarity ECL developing mixture, wrapped in plastic film and developed on photo paper. Protein expression was analyzed semi-quantitatively by densitometry of captured gel images using Fiji.

Immunocytochemistry

Dermal fibroblasts were cultured on glass coverslips, fixed in 4% paraformaldehyde for 15 minutes and washed in PBS. Following permeabilization in 0.1% triton-X in PBS for 15 minutes at RT and washing in PBS, samples were blocked in 0.1% BSA in PBS at RT for 2 hours. The primary antibodies were diluted in 0.1% BSA and added to the samples for

overnight incubation at 4°C. After washing in PBS five times, samples were incubated with secondary antibodies (in 0.1% BSA) at 4°C overnight in the dark, followed by three washes in PBS and one in distilled water. Coverslips were mounted onto glass microscope slides using ProLong Diamond mounting medium (Thermofisher Scientific, #P36961) and left to set overnight at RT in the dark. Hoechst and pahlloidin staining were used as structural dyes. A list of primary and secondary antibodies used is found in Supplemental Table 1.

Sucrose density gradients

Briefly, fibroblasts (60% - 80% confluence) were treated with cyclohexamide (CHX) at final concentration of 100 μg/ml for 15 min at 37 ° C before harvesting. After washing in cold PBS (with CHX, 100 μg/ml) cells were lysed in lysis buffer (20 mM HEPES at pH 7.4, 50 mM KCl, 5 mM $Mg(OAC)_2$, 0.5 % [v/v] IGEPAL® CA-630 (Sigma), 0.5 % [w/v] deoxycholate (Sigma), 100 μg/mL cycloheximide (Sigma) with complete EDTA-free protease inhibitors (Roche) and 500 U/mL RNase inhibitor (RNaseOUTTM, Invitrogen). After incubating 15 min on ice, cell lysates were cleared in a microfuge at 20,000 g for 10 min at 4 °C. Equal amounts (typically 1.0 $A_{254\ nm}$ unit) of sample were loaded onto 5 % – 45 % (w/v) sucrose gradients in 14 mL of gradient buffer (20 mM HEPES pH 7.4, 50 mM KCl, 5 mM $Mg(OAC)_2$, 100 μg/ml CHX) with complete EDTA-free protease inhibitors (Roche) and centrifuged (Beckmann SW40 rotor at 284,600 g for 2.5 hr at 4°C). Sucrose gradients were prepared using a Biocomp Gradient Master according to the manufacture's manual. Samples were unloaded using a Brandel gradient fractionator and the polysome profiles detected using an ÄKTAprime Plus system (GE Healthcare).

Measurement of protein synthesis

OP-Puro (Invitrogen; final concentration 50 μM) was added to the culture medium (Dulbecco's Modified Eagle Medium (DMEM, GibcoTM GlutaMAXTM), 10 % fetal bovine serum (Sigma) and 1 % Penicillin- Streptomycin (Pen-Strep, Sigma)) for 60 min. Cells were

removed from wells and washed twice in ice-cold PBS with 100 μg/ml cycloheximide. Cells were fixed and permeabilized using the Cytofix/Cytoperm Fixation Permeabilization Kit (BD Biosciences). Azide-alkyne cycloaddition was performed using the Click-iT Cell Reaction Buffer Kit (Invitrogen) with azide conjugated to Alexa Fluor 488 at 5 μM final concentration. Following the 30 min reaction, cells were washed twice in PBS and then resuspended in PBS supplemented with 2% fetal bovine serum and analyzed by flow cytometry (Becton Dickinson LSR Fortessa analyzer). Flow cytometry data analysis was performed using FlowJo v10.1 (FlowJo, Ashland, OR). Relative rate of protein synthesis was calculated by normalizing OP-Puro signals to control cells after subtracting background fluorescence (cells without OP-Puro incorporation).

Generation of the *rpl13* mutant CRISPR-Cas9 zebrafish model

The synthesis of the specific oligonucleotides (Eurofins Genomics, Vimodrone, Italy) and the preparation of gRNA were carried out as previously described[(4)]. The gRNA template, upon subcloning in the pT7-gRNA plasmid (Addgene), was in vitro transcribed using MEGAshortscript T7 kit (Invitrogen) and purified with mirVana miRNA Isolation Kit (Invitrogen). The size and quality of the resulting gRNA were confirmed by electrophoresis on a 10% (v/v) polyacrylamide-urea-SDS gel.

For the Cas9 mRNA *in vitro* transcription, the pT3TS-nCas9n vector (Addgene, #46757) was linearized by XbaI (New England BioLabs, Ipswich, Massachusetts, USA) digestion and purified using the Nucleospin Gel and PCR Clean-up Kit (Macherey-Nagel, Düren, Germany). DNA was transcribed using mMESSAGE mMACHINE T3 Kit (Invitrogen, Carlsbad, California, USA). mRNA polyadenylation was performed using the Poly(A) Tailing Kit (Ambion, Waltham, Massachusetts, USA) and the Cas9 transcript was purified by RNeasy Mini Kit (Qiagen, Hilden, Germany)[(4)]. The mRNA quality was checked by electrophoresis on 1% (w/v) formaldehyde agarose gel. The gRNA (12.5 ng/μL) and Cas9 mRNA (300 ng/μL)

were mixed in Danieau solution (58 mM NaCl, 0.7 mM KCl, 0.4 mM $MgSO_4$, 0.6 mM $Ca(NO_3)_2$, 5 mM Hepes, pH 7.6) with a tracer dye (0.5 mg/mL, dextran conjugated with tetramethylrhodamine, Molecular Probes, Carlsbad, California, USA) in a final volume of 5 μL and pre-heated at 60°C for 10 min. Microinjection was carried out using an InjectMan micromanipulator (Eppendorf, Hamburg, Germany) assembled on a Leica M165 FC stereomicroscope. Injection pressure and time were modulated to calibrate the injected volume, ranging from 2 to 4 nL per embryo. Mosaic fish were screened by T7 endonuclease assay, briefly after 24 hours the DNA from single injected embryos was extracted by proteinase K digestion (2.5 mg/mL, Sigma Aldrich, Darmstadt, Germany) in lysis buffer (100 mM Tris HCl, pH 8.5, 5 mM EDTA, 0.2% (w/v) SDS, 200 mM NaCl) o/n at 55°C, followed by isopropanol precipitation and resuspension in 20 mM Tris-HCl, 1 mM EDTA, pH 8.0. DNA was PCR amplified using the following primers: *rpl13* sense 5'-GTGTACATGCATTGTCGTTGACT-3' and reverse 5'-GATTCCAGGAAAGCAGAATTTTT-3' spanning the target sequence. The PCR amplicon (10 μL) underwent a denaturing/annealing cycle consisting of 5 min at 94°C, followed by cooling to 85°C, at −2°C per sec and further to 25°C, at −0.1°C per sec, and was finally digested with 0.2 U/μL T7 endonuclease I (New England BioLabs) at 37°C for 1 h. The sample was run on 8% (v/v) polyacrylamide gel. The targeting was then confirmed by Sanger Sequencing. The mutation allele in heterozygous ($rpl13^{+/L191Lfs}$) and homozygous ($rpl13^{L191Lfs/L191Lfs}$) zebrafish was determined by DNA extraction from tail clip of adult zebrafish, followed by Sanger sequencing.

Quantitative PCR (qPCR) and Western immunoblotting (WB) in zebrafish

RNA was extracted from adult WT and $rpl13^{L191Lfs/L191Lfs}$ skin (n=3 per genotype) using Qiazol (Qiagen) following manufacturer's instructions. RNA quantity was determined by NanoDrop spectrophotometer and RNA quality by agarose gel electrophoresis. cDNA was synthesized

using the High Capacity cDNA Transcription kit (Applied Biosystems) according to manufacturer's protocol in a final volume of 20 μL. qPCR for *rpl13* and *dna15ta1* was performed in 25 μL reaction mixtures with 12.5 μL SYBR Green Master mix (Applied Biosystems) using the QuantStudio 3 thermocycler and the QuantStudio Design & analysis software (Applied Biosystems). The following primers were used: for *rpl13* forward, 5'-GGACCAGTCATGCCCATCAA-3' (nt 466-485) and reverse, 5'-TTCTTCTCGACGTCCTGCTC-3' and for *dna15ta1* forward, 5'-TACTGTGCTCAAATTGCTTCA-3' and reverse, 5'-AATGAGTACTGTGAACTTAATCCAT-3'. The annealing temperature was 60 °C and samples were run in triplicate. $\Delta\Delta$Ct was used for quantitation.

Protein extract was obtained from adult WT and $rpl13^{L191Lfs}/^{L191Lfs}$ soft tissue (n=3 per genotype) as described in the paper by Tonelli et al.[36]. For each sample 30 μg were run on 4-20% Mini-PROTEAN TGX Gel (Bio-Rad). Expression of eL13 was evaluated by WB using a primary antibody against eL13 (RPL13 Polyclonal Antibody, Invitrogen, #PA5-41715, 1:500 dilution) and a HRP-conjugated anti-rabbit secondary antibody (Anti-rabbit IgG, Cell Signaling, #7074S 1:10000 dilution) according to standard procedures. The signal was detected by Westar Supernova ECL western reagent (Cyanagen) and images were acquired and analysed with ImageQuant LAS 4000 (GE Healthcare), using the ImageQuant LAS 4000 1.2 software.

Supplemental Table S1. Antibodies and immunocytochemistry reagents

**Ab/probe name**	**Company, code**	**Type**	**Host species**	**Reactivity**	**ICC concentration/dilution**
RPL13	Santa Cruz, #sc-100829	primary, monoclonal	mouse	mouse, rat and human	2 μg/ml
RPL7	Sigma, #HPA058373	primary, polyclonal	rabbit	mouse, human	1 μg/ml
RPL28	Sigma, #HPA050459	primary, polyclonal	rabbit	human	2.5 μg/ml
RPS19	Abcam, #ab40833	primary, polyclonal	goat	human	10 μg/ml
Calnexin	Abcam, #ab192439	primary, polyclonal	goat	mouse, rat, dog, human, african green monkey	4 μg/ml
Goat anti-Mouse IgG	Thermofisher Scientific, #A32723	secondary, conjugated Alexa Fluor™ 488	goat	mouse	1:250
Donkey anti-Mouse IgG	Thermofisher Scientific, #A10037	secondary, conjugated Alexa Fluor™ 568	donkey	mouse	1:250
Donkey anti-Goat IgG	Thermofisher Scientific, #A11055	secondary, conjugated Alexa Fluor™ 488	donkey	goat	1:300
Phalloidin	Thermofisher Scientific, #A22287	conjugated Alexa Fluor™ 647	X	X	1:40
Hoechst	Thermofisher Scientific, #H3570	Hoechst 33342	X	X	1:3000

ICC= immunocytochemistry

Supplemental references

1. Costantini A, Skarp S, Kampe A, Makitie RE, Pettersson M, Mannikko M, et al. Rare Copy Number Variants in Array-Based Comparative Genomic Hybridization in Early-Onset Skeletal Fragility. Front Endocrinol (Lausanne). 2018;9:380.

2. Costantini A, Valta H, Baratang NV, Yap P, Bertola DR, Yamamoto GL, et al. Novel fibronectin mutations and expansion of the phenotype in spondylometaphyseal dysplasia with "corner fractures". Bone. Apr 2019;121:163-71.

3. Doyard M, Bacrot S, Huber C, Di Rocco M, Goldenberg A, Aglan MS, et al. FAM46A mutations are responsible for autosomal recessive osteogenesis imperfecta. J Med Genet. Apr 2018;55(4):278-84.

4. Jao L-E, Wente SR, Chen W. Efficient multiplex biallelic zebrafish genome editing using a CRISPR nuclease system. Proceedings of the National Academy of Sciences of the United States of America. 2013;110(34):13904-9.
